# Impact of traditional risk factors for the outcomes of atrial fibrillation across race and ethnicity and sex groups

**DOI:** 10.1016/j.ijcha.2020.100538

**Published:** 2020-05-29

**Authors:** Timothy Shih, Karina Ledezma, Mark McCauley, Jalees Rehman, William L. Galanter, Dawood Darbar

**Affiliations:** a Departments of Medicine, University of Illinois at Chicago, USA; b Departments of Pharmacology, University of Illinois at Chicago, USA; c Department of Medicine, Jesse Brown Veterans Administration, Chicago, IL, USA

**Keywords:** Atrial fibrillation, Risk factors, Racial disparities, Stroke, Sex disparities

## Abstract

**Background:**

Although traditional risk factors for atrial fibrillation (AF) and its outcomes are established in whites, their role in the pathogenesis of AF across race-ethnicity and both sexes remain unclear. Cohort studies have consistently shown worse AF-related outcomes in these groups. The objective of this study was to determine the role played by race- and sex-specific risk factors in AF outcomes in non-Hispanic blacks (NHBs), Hispanics/Latinos (H/Ls), and non-Hispanic whites (NHWs).

**Methods:**

Using electronic health records (EHR), 3607 patients with an ICD-9 code for AF were identified over a 7-year period. Risk factors were identified from ICD to 9 CM claims data: hypertension (HTN), type 2 diabetes mellitus (T2DM), stroke/transient ischemic attack (TIA), smoking, chronic obstructive pulmonary disease (COPD), coronary artery disease (CAD), peripheral arterial disease (PAD) and obstructive sleep apnea (OSA). Multivariate analysis of variance was used to compare the incidence of AF risk factors.

**Results:**

NHBs and H/Ls with AF experienced more stroke than NHWs (27% and 24% vs. 19% P < 0.01). Females had less HTN (48.4% vs 51.6% [males], P = 0.0002), CAD (47.4% vs 55.7% [males], P = 0.02), and smoking rates (38.2% vs 61.8% [males], P < 0.0001) but higher stroke rates (25.9% [female] vs 21.8% [males], P < 0.0001). Age-adjusted risk factors for stroke varied markedly across race-ethnicity and sex.

**Conclusions:**

We identified differences in risk factors for AF and stroke across race-ethnicity and sex. The findings of our study are hypothesis generating and should be used to direct future studies.

## 1 Introduction

Atrial fibrillation (AF), the most common cardiac arrhythmia in clinical practice, affects between 2.7 and 6.1 million people in the U.S and is projected to increase to 12.1 million by 2030 [1], [2], [3]. Importantly, AF confers a significant risk for the development of stroke, which increases based on a patient’s cardiovascular risk factor profile [4] While it is reported that non-Hispanic whites (NHWs) experience more AF than non-Hispanic Blacks (NHBs) and Hispanics/Latinos (H/Ls), subjects of African and Hispanic descent carry a greater burden of AF-related risk factors and thus higher rates of stroke [5], [6]. Compared with NHWs, H/Ls are more likely to be younger at AF onset and female with higher AF-related stroke [7]. They are also disproportionately affected by hypertension (HTN) and type 2 diabetes mellitus (T2DM) compared to NHWs [8], [9], [10]. Similarly, NHBs have a greater burden of cardiovascular risk factors including HTN, T2DM, left ventricular hypertrophy, increased body mass index (BMI), and tobacco use as compared to NHWs [10], [11].

The rates of traditional risk factors for AF and stroke represent 58% of the burden of AF among blacks as compared to 44% in whites [11], [12]. This results in an AF paradox in NHBs, where higher rates of AF-related risk factors lead to a lower incidence of AF compared to NHWs but higher rates of AF-related stroke. The mechanism behind this ‘double paradox’ is poorly understood [12]. Nevertheless, there is convincing evidence that ethnic-specific risk factors influence the development of AF differently. Data from the Multi-Ethnic Study of Atherosclerosis (MESA) showed that HTN is major contributor to the higher percentage of AF in NHW minorities [13]. This study reported that the population attributable fraction (fraction of modifiable risk factors for AF in a given period attributable to the incidence of AF) for HTN was twice as high in H/Ls and two-thirds that of NHBs compared to NHWs. Accordingly, HTN appears to be less influential on AF incidence in NHWs than in NHBs and H/Ls. However, it remains unclear what impact if any ethnic- and sex-specific differences in risk factors have on AF outcomes. The poorer outcomes in NHBs and H/Ls with AF highlight the need for better understanding of traditional risk factors that disproportionately affect these vulnerable populations with AF. Thus, the major goal of this study was to examine the role of race- and sex-specific differences in traditional risk factors implicated in the outcomes of AF using a multiethnic cohort of NHWs, NHBs, and H/Ls.

## 2 Methods

### 2.1 Study cohort

The study population consisted of NHBs, H/Ls, and NHWs (based on self-reported race or ethnicity) aged ≥ 18 years with more than one *International Classification of Diseases, Ninth Revision* (ICD 9) Clinical Modification (CM) code 427.3 primary diagnosis of AF upon discharge from the University of Illinois Hospital (UIH) between January 1, 2008 and September 30, 2015. Patients without documented race or ethnicity or with race or ethnicity that was other than NHB, H/L, or NHW were excluded from the analysis.

Variables reported at baseline included: race or ethnicity, age at first presentation with AF during the study period, HTN, T2DM, congestive heart failure (CHF), coronary artery disease (CAD), peripheral arterial disease (PAD), obstructive sleep apnea (OSA), smoking history, chronic obstructive lung disease (COPD), and stroke or transient ischemic attack (TIA). The presence of these risk factors was determined based on ICD-9 CM codes upon discharge from UIH between January 1, 2008 and September 30, 2015. Estimated yearly stroke risk was calculated using the CHA_2_DS_2_-VASc score from these patient specific risk factors.

### 2.2 Definitions

At our institution, we defined arterial HTN by a history and/or the presence of antihypertensive therapy. Criteria for CAD include a history of myocardial infarction (MI) or typical angina, previous coronary bypass surgery or percutaneous coronary intervention (PCI) and drug treatment. CHF is defined by a history and/or drug treatment for heart failure. We define T2DM as having impaired fasting glycemic levels above normal range (HbA1c 6.5% or higher), a fasting plasma glucose level of 126 mg/dL or a random plasma glucose of 200 mg/dL or higher. OSA is defined by a positive sleep study or when a patient receives continuous positive airways pressure therapy. The presence of AF risk factors was solely based on ICD-9 CM codes.

### 2.3 Validation cohort

We sampled 508 patients from the total AF cohort and performed manual review of their charts. This was to not only ensure that the sampled cohort were representative of the total population but also so that sensitivity analysis can be performed to assess the diagnostic accuracy of the algorithm used to identify patients with a history of AF in our electronic health record (EHR).

### 2.4 Statistical analysis

Categorical data was reported as frequencies and proportions using Chi-Square test, which was also used to examine proportional differences across all three racial or ethnic groups (NHB, NHW, and H/L). Significant differences in frequency (proportion) between each group are underscored by P < 0.05. Analysis of variance (ANOVA) was used to compare differences between means of continuous variables. Wilcoxon Rank sum test was used to determine median age of AF onset in the entire sample population. Sensitivity analysis was used to generate a positive predictive value (PPV) measuring the accuracy of our algorithm in extracting AF cases from the UIH EHR.

**Multivariate logistic regression** was used to compute AF odds ratios (OR) and corresponding 95% confidence intervals (CIs) to measure the association between stroke and participant demographics, including traditional risk factors within each racial or ethnic group. All multivariate models were adjusted for age, sex, and the risk factor (independent variable) being analyzed e.g., HTN. Analyses were performed separately for NHBs, H/Ls and NHWs to assess for sex differences, as well as across racial- and sex-specific groups combined. All statistical analyses were performed with *SAS v9.4* (SAS Inc., Cary, NC) and set at *a-priori* with *ɑ*-level of 0.05 to denote statistical significance.

## 3 Results

### 3.1 Study cohort

**Fig. 1:**
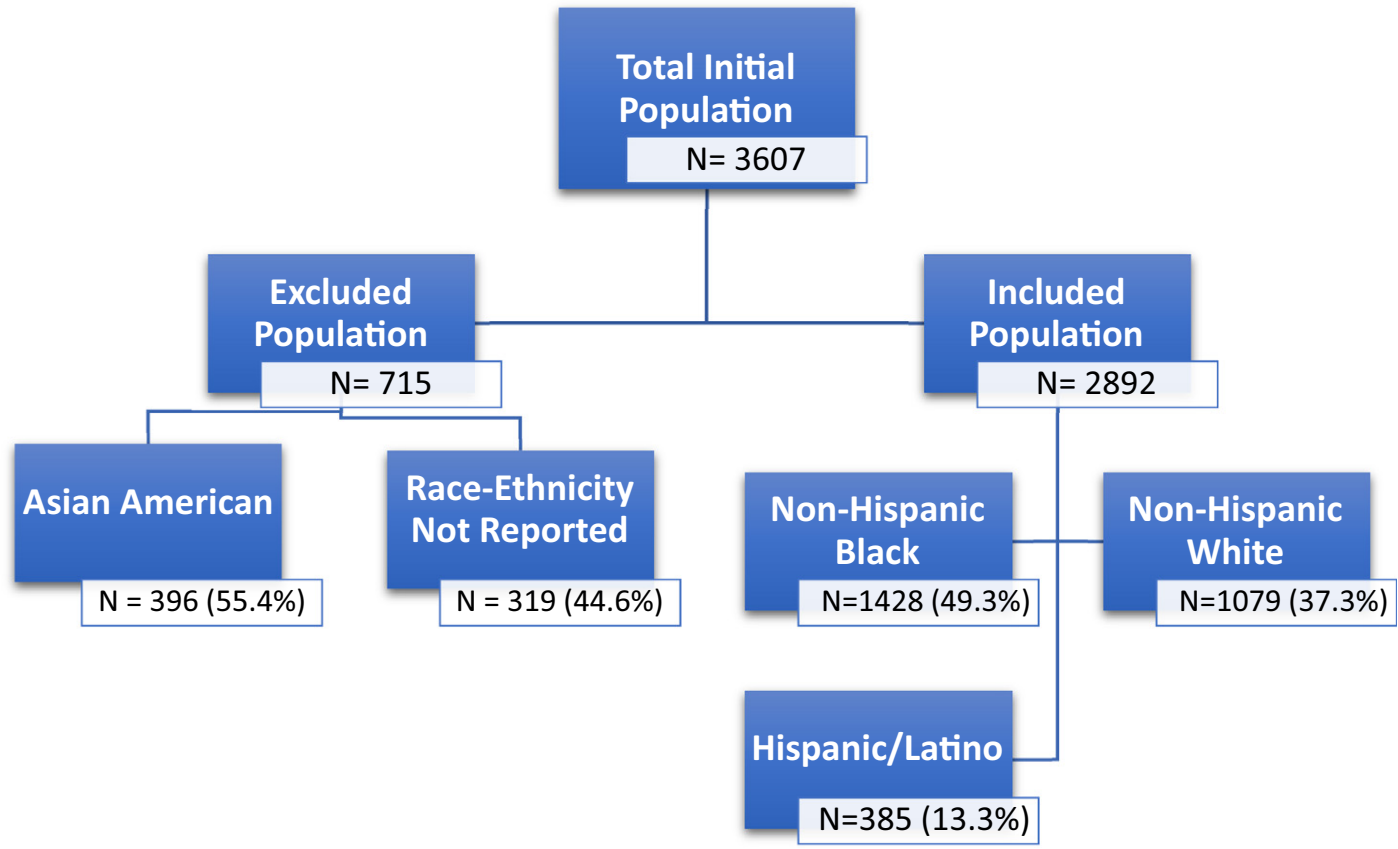
A flow diagram illustrating the recruitment of a multi-ethnic cohort.

A total of 3607 patients were identified with greater than or equal to 1 diagnosis of AF based on ICD-9 CM 427.3 upon discharge from UIH from 2008 through September 2015. Given the populations of interest, 715 patients were excluded from the study based on a self-reported race-ethnicity outside of our target populations (Fig. 1). The final study cohort consisted of 2892 patients, of which 28 (49.3%) were NHB, 1079 (37.3%) NHWs, and 385 (13.3%) H/Ls. Overall, 46.8% of the patients were female, though this proportion varied significantly across racial and ethnic groups: 39.9% (NHWs) vs. 51.6% (NHBs; P < 0.001) vs. 48.1% (H/Ls; P < 0.01). The median age at first diagnosis was 67 years with NHBs representing the youngest subgroup (66 years (NHBs) vs 69 years (NHWs) vs 68 years (H/Ls, P < 0.0001).

### 3.2 Risk factors for AF

As shown in Table 1, there was a high prevalence of HTN in the study cohort (81.4%) but the prevalence was significantly higher among NHB and H/L patients as compared to NHWs. In addition, there was a greater prevalence of T2DM, PAD, and CHF among NHBs and H/Ls when compared to NHWs, giving rise to higher mean CHA2DS2-VASc scores. In addition, NHB and H/L patients had higher rates of stroke as compared to NHWs. NHBs had higher rates of OSA and smoking when compared to both NHWs and H/Ls. In contrast, H/Ls had lower rates of COPD as compared to both NHW and NHBs. Risk factors for AF and CHA2DS2-VASc scores varied significantly by race-ethnicity (Table 1) and **Supplementary** Table 1 presents the CHA2DS2-VASc scores as proportions of patients per score.

**Table 1:** Traditional risk factors in our multi-ethnic cohort of atrial fibrillation (AF).

	Total	Non-Hispanic Whites	Non-Hispanic Blacks	Hispanic/Latinos
	Frequency (%)			
HTN	2355 (81.4%)	816 (75.6%)	1214 (85.0%)^ǂ^	325 (84.4%)^ǂ^
T2DM	1052 (36.4%)	307 (28.5%)	563 (39.4%)^ǂ^	182 (47.3%)^ǂ^
OSA	218 (7.5%)	49 (4.5%)	144 (10.1%)^ǂ^	25 (6.5%)
PAD	1301 (45.0%)	403 (37.3%)	690 (48.3%)^ǂ^	208 (54.0%)^ǂ^
COPD	511 (17.7%)	194 (18.0%)	271 (19.0%)	46 (11.9%)^ǂ^
Smoking	979 (33.9%)	303 (28.1%)	569 (39.8%)^ǂ^	107 (27.8%)
CHF	47 (1.6%)	5 (0.5%)	35 (2.5%)^ǂ^	7 (1.8%)^ǂ^
Stroke/TIA	685 (23.7%)	207 (19.2%)	384 (26.9%)^ǂ^	94 (24.4%)^ǂ^
CHA_2_DS_2_-VASc^ƒ^	3.48	3.17	3.61	3.81

ǂ P < 0.05; ƒ mean.

Abbreviations: CHA_2_DS_2_-VASc^ƒ^, congestive heart failure, hypertension, age, diabetes, stroke, vascular, sex; CHF, congestive heart failure; COPD, chronic obstructive pulmonary disease; HTN, hypertension; OSA, obstructive sleep apnea; PAD, peripheral arterial disease; T2DM, Type II diabetes mellitus; TIA, transient ischemic attack

### 3.3 Association of traditional AF risk factors with stroke

The burden of AF risk factors was similar between H/Ls and NHBs that significantly increased the risk of stroke when compared to NHWs with the same comorbidities (Table 2). Furthermore, H/Ls with HTN were 3.1 times more likely to suffer a stroke as compared to those without HTN (OR 3.1, 95% CI 1.28 to 7.58, P = 0.01), while NHBs were at 2.0-fold increased risk in the presence of HTN (OR 2.0, 95% CI 1.33 to 2.88, P = 0.001). T2DM also increased the risk of stroke in both H/Ls (OR 1.7, 95% CI 1.05 to 2.70, P = 0.03) and NHBs (OR 1.34, 95% CI 1.06 to 1.70, P = 0.02). Surprisingly, HTN and T2DM were not significant contributors to the risk of stroke in NHWs in our study population.

**Table 2:** Association of AF risk factors with stroke by race or ethnicity.

		Non-Hispanic Whites			Non-Hispanic Blacks			Hispanic/Latino	
		Stroke			Stroke			Stroke	
	OR	95% CI	P value	OR	95% CI	P value	OR	95% CI	P value
HTN	1.3	0.92–1.94	0.13	2.0	1.33–2.88	0.001^‡^	3.1	1.28–7.58	0.013^‡^
T2DM	1.1	0.81–1.58	0.46	1.3	1.06–1.70	0.016^‡^	1.7	1.05–2.71	0.029^‡^
Smoking	1.5	1.04–2.03	0.028^‡^	1.4	1.07–1.73	0.011^‡^	1.8	1.08–2.97	0.024^‡^
CAD	1.5	1.07–1.99	0.018^‡^	1.8	1.44–2.32	<0.0001^‡^	1.5	0.95–2.47	0.08
PAD	1.9	1.14–3.08	0.013^‡^	1.8	1.29–2.59	0.0007^‡^	2.6	1.40–4.90	0.003^‡^
VD	1.5	1.13–2.09	0.007^‡^	1.9	1.49–2.41	<0.0001^‡^	1.7	1.07–2.83	0.025^‡^
COPD	1.4	0.98–2.07	0.061^‡^	1.3	0.97–1.74	0.08	3.3	1.73–6.19	0.0003^‡^
OSA	0.7	0.28–1.62	0.38	1.2	0.77–1.69	0.51	1.6	0.66–3.84	0.3
CHF	1.2	0.13–10.73	0.89	1.8	0.88–3.59	0.107	1.2	0.23–6.30	0.83
CHA_2_DS_2_-VASc	6.8	5.27–8.87	<0.0001^‡^	7.1	5.74–8.82	<0.0001^‡^	7.2	4.70–11.10	<0.0001^‡^

^‡^P < 0.05. Abbreviations as for Table 1.

PAD was a significant risk factors for stroke across all 3 racial and ethnic groups contributing to 2.6-fold increased risk for stroke in H/Ls (OR 2.62, 95% CI 1.40 to 4.90, P = 0.003), 1.9-fold risk in NHWs (OR 1.9, 95% CI 1.14 to 3.08, P = 0.01), and 1.8 fold increased risk in NHBs (OR 1.83, 95% IC 1.29 to 2.59, P = 0.0007). As for smoking, H/Ls were at 1.8 fold increased risk for stroke (OR 1.8, 95% CI 1.08 to 2.96, P = 0.02), NHBs 1.4 times (OR 1.4, 95% CI 1.07 to 1.73, P = 0.01), and NHW patients were 1.5 times more likely (OR 1.5, 95% CI 1.04 to 2.03, P = 0.03). CAD increased stroke risk in NHBs (OR 1.83, 95% CI 1.44 to 2.30, P < 0.0001) and NHWs (OR 1.5, 95% 1.06 to 1.99, P = 0.02) without affecting stroke risk in H/Ls. In contrast, COPD accounted for a 3.3-fold higher stroke risk in H/Ls (OR 3.27, 95% CI 1.73–6.18, P = 0.0003) with no impact on stroke development in NHBs and NHWs. Furthermore, higher CHA2DS2-VASc score was correlated with higher stroke risk among all races (Table 2).

### 3.4 Traditional AF risk factors by sex

There were also marked differences in AF risk factors by sex. Overall, females had significantly lower rates of HTN, CAD and smoking than men (Fig. 2a, Fig. 2b, Fig. 2c). However despite this, females experienced significantly higher rates of stroke and CHA2DS2-VASc scores. Among NHBs, females had more HTN. Fig. 2a shows that NHB females were significantly more likely to develop a stroke given a history of CAD, PAD or COPD. Conversely, NHB males had an increased risk of stroke if they had HTN, T2DM, a smoking history, CAD or PAD.

**Fig. 2a:**
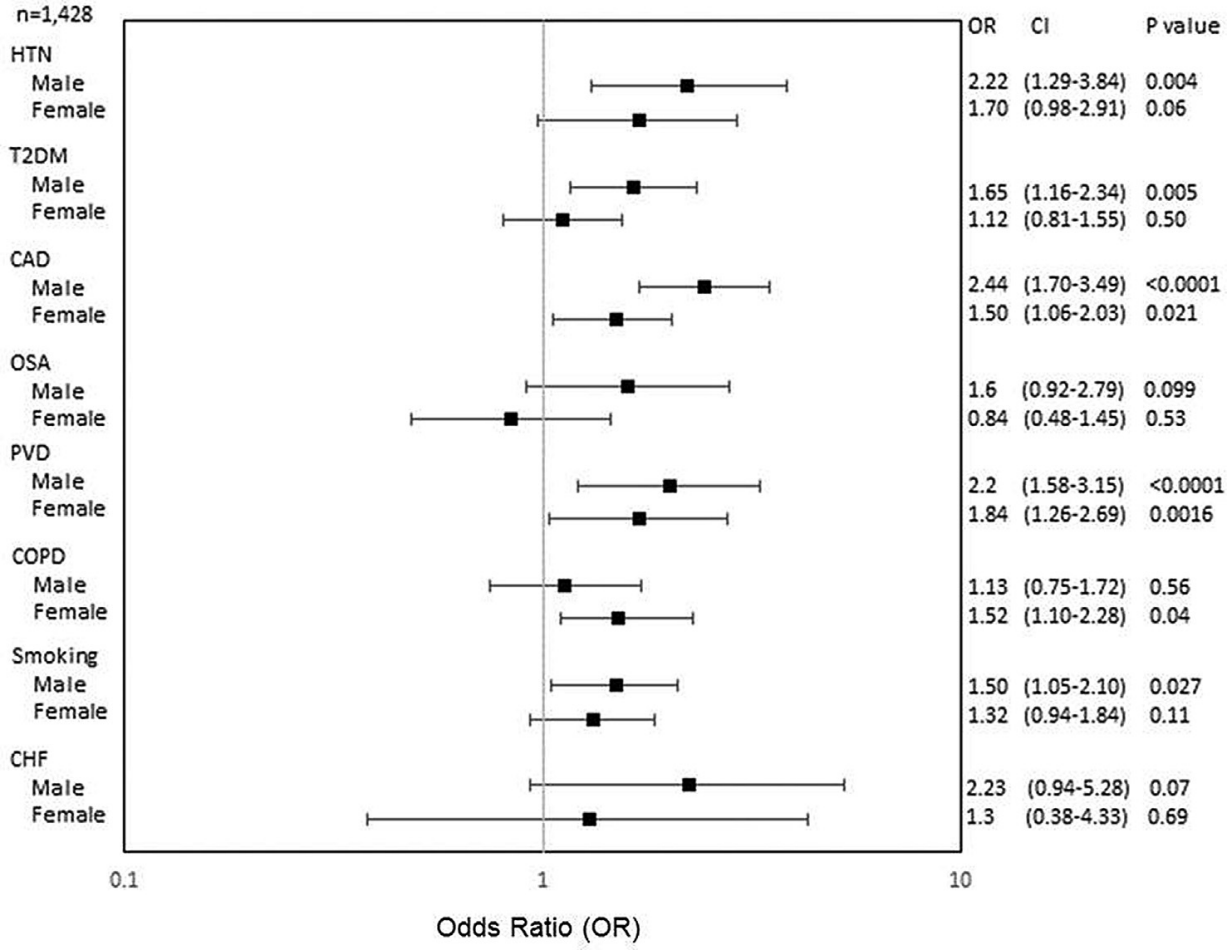
Impact of age-adjusted AF risk factors on stroke in non-Hispanic blacks (NHBs). Abbreviations: CI, confidence interval; HTN, hypertension; T2DM, type II diabetes mellitus; OSA, obstructive sleep apnea; COPD, chronic obstructive pulmonary disease; CHF, congestive heart failure; TIA, transient ischemic attack.

**Fig. 2b:**
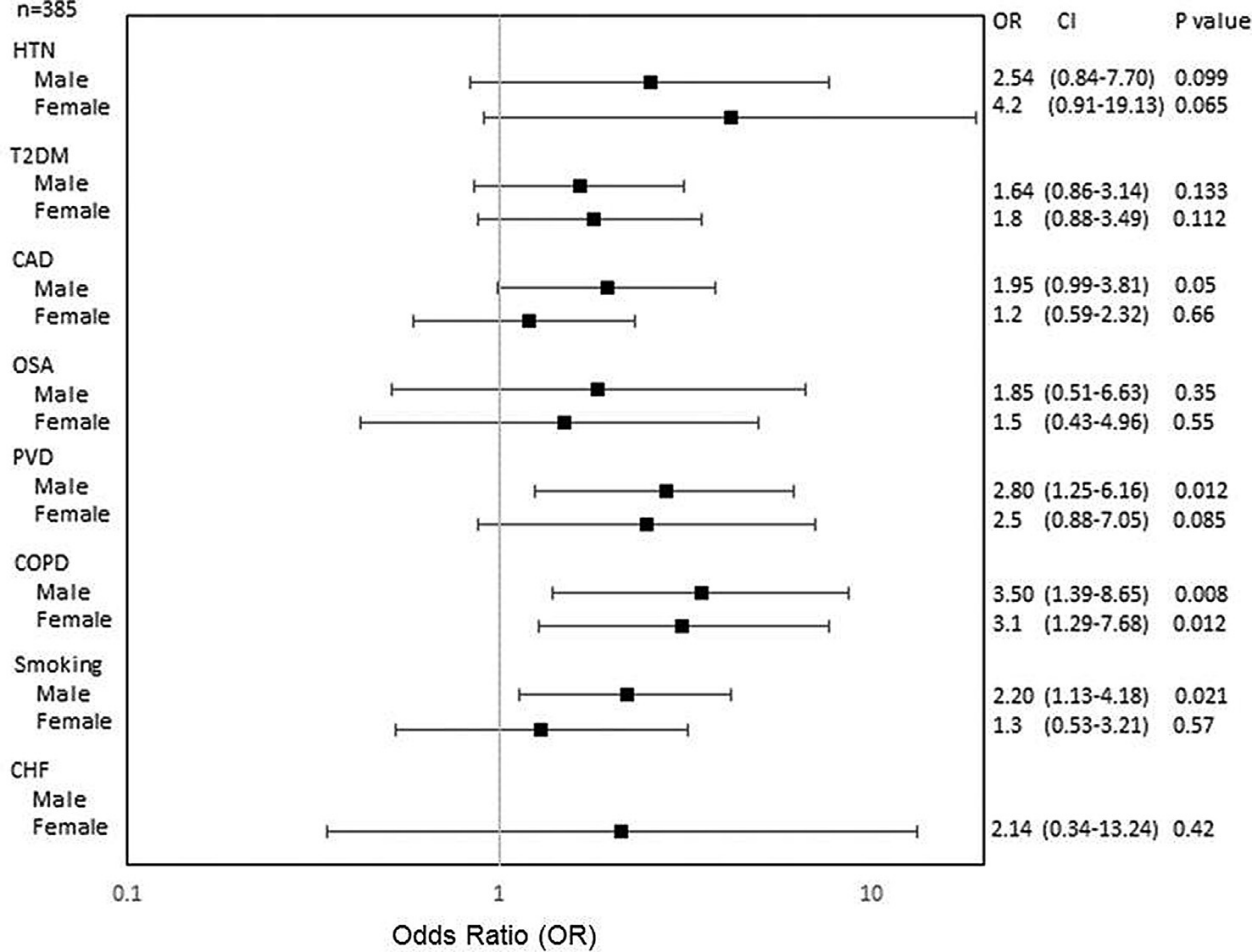
Impact of age-adjusted AF risk factors on stroke in Hispanics/Latinos (H/Ls). Abbreviations as in Fig. 2a.

**Fig. 2c:**
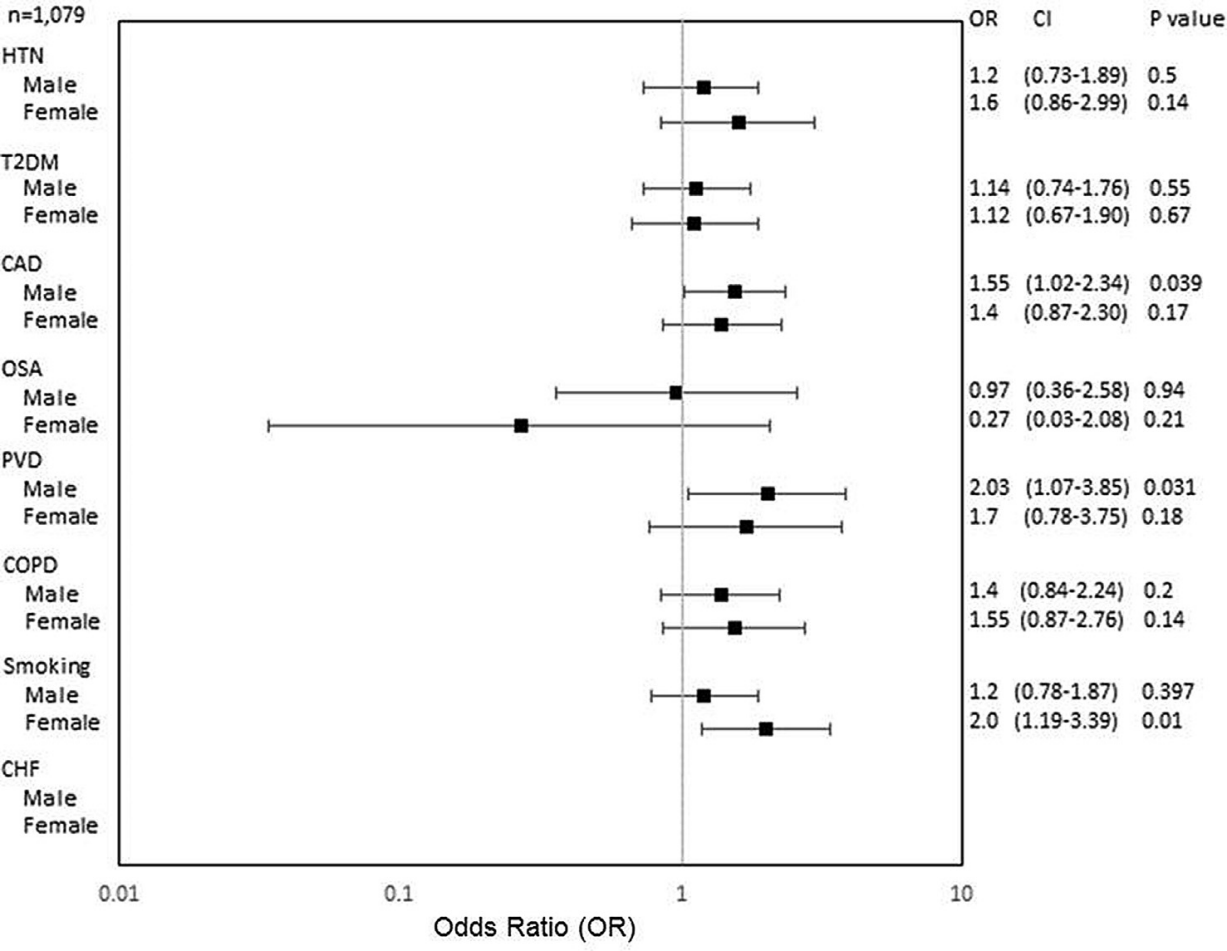
Impact of age-adjusted AF risk factors on stroke in Non-Hispanic Whites (NHWs). Abbreviations as in Fig. 2a.

Fig. 2b shows that among H/Ls, females with COPD had a 3.1-fold higher risk of developing a stroke as compared with those without COPD. Hispanic females were also 4.2 times more likely to develop stroke if they had HTN compared to those without HTN, although the difference was borderline significant. In contrast, H/L males had a 3.5 times likelihood of having a stroke with COPD versus no COPD; a 2-fold increased risk of having a stroke with CAD verses no CAD and a smoking history; and a 2.8-fold risk of having a stroke with PAD vs no PAD.

As for NHWs, males with PAD were at 2-fold increased risk of stroke and females who smoked were twice as likely to have a stroke compared to non-smokers (Fig. 2c).

### 3.5 Validation cohort

The cohort of 508 patients comprised of 34% NHB, 34% H/L and 32% NHW (see Table 3). Majority were female (51%), had an average age at AF encounter of 67 years, and a language preference of English (80%) followed by Spanish (18.8%). Majority of NHBs had paroxysmal AF (39%) compared to a higher proportion of H/Ls with persistent AF (51%), whereas NHWs showed an even distribution of paroxysmal, persistent, and permanent type of AF (26%, 29.6%, and 29.4%, respectively, P < 0.0001). In terms of socio-demographics, H/Ls and NHBs had the lowest income (both 37%) while NHWs were mainly from middle and high-income backgrounds (p < 0.0001). Although NHWs were referred for ablation procedures for their AF significantly more than their NHB and H/L counterparts (11% vs 2% vs 5%, P = 0.005), there were no racial differences in anticoagulation use: 66% of H/Ls, 57% of NHBs, and 60% of NHWs were anticoagulated appropriately (p = 0.29). Compared to NHWs and NHBs, H/Ls had statistically significantly higher rates of DM type 2, HTN, pulmonary HTN, and valvular disease (P < 0.0001). NHBs and H/Ls had significantly higher number of admissions compared to NHWs (p < 0.0001). Although nonsignificant, NHBs had the highest rates of preexisting stroke (22% vs 18% H/Ls and 16% NHW, P = 0.34), yet H/Ls had the highest stroke incidence after a diagnosis of AF (18% vs 13% in both NHW and NHB; P = 0.29). In this small validated study, H/L showed the highest disparities in ethnic-specific stroke risk factors, adverse outcomes (death, major bleeding, hospital admission), and a trend towards significantly higher incidence of AF-related stroke.

**Table 3:** Clinical characteristics of the validation cohort (N = 508).

Frequency *(%)*	Non-Hispanic White	Hispanic/Latino	Non-Hispanic Black	*P value*
	*N = 134*	*N = 144*	*N-144*	
**Risk Factors**				
T2DM	35 (26.1)	74 (51.4)	59 (40.9)	<0.0001
HTN	105 (78.4)	129 (89.6)	121 (84)	0.004
Pulmonary HTN	9 (6.7)	23 (16.2)	20 (13.9)	0.046
Valvular heart disease	56 (43.8)	78 (54.9)	46 (33.6)	0.002
CHA_2_DS_2_-VASc ≥ 2	108 (80.6)	135 (93.8)	131 (90.9)	0.001

**Socioeconomic Status**				
High	1 (1)	<1%	<1%	
Middle	70 (52)	13 (9)	18 (13)	
Low	35 (26)	53(37)	53 (37)	<0.0001
Unknown	28 (21)	77 53)	72 (49)	

**AF Type**				
Paroxysmal	35 (26)	73 (51)	56 (39)	<0.0001
Persistent	39.6 (29.6)	23 (16)	12 (8)	
Permanent	39.4 (29.4)	29 (20)	6 (4)	
Unknown	20 (15)	19 (13)	70 (49)	

**Oral Anticoagulation**	81 (60.5)	95 (66)	81 (57)	0.29
Aspirin alone	39 (39.4)	29 (39.7)	37 (43)	0.47

Socioeconomic (SES) parameters were described as mean per capita income, stratified into three categorical variables: low <$30,000), middle ($30–70,000), high (>$70,000). The mean per capita income was collected from residency zip codes in the EMR using data from Chicago Census Data. P < 0.05 determines statistical significance.

### 3.6 Discussion

This study demonstrated important differences in the prevalence of race- and sex-specific risk factors for AF and risk of stroke. Our patients of African and Hispanic descent experienced significantly more stroke when compared to European whites. The presence of either HTN or T2DM increased the risk of stroke in H/Ls and NHBs compared to those without these risk factors. Interestingly, neither of these risk factors influenced the risk of stroke in NHWs. This emphasizes the need to investigate both traditional risk and protective factors across race-ethnicity that drive their stroke rates compared to NHWs. Our findings are hypothesis generating and should be used to direct future studies.

Smoking and PAD were influential in increasing the risk of stroke across all 3 racial and ethnic groups. Aside from underlying genetic factors, SES, and environmental determinants of health, disparities in AF-related comorbidities can potentiate risk for stroke and other AF-related outcomes that need to be understood for risk factor control and secondary stroke prevention among ethnic minority populations.

The incidence of AF is lower among NHBs than NHWs but AF confers an increased risk of all adverse outcomes in NHBs, termed the AF paradox [5], [12]. This is supported by our data which shows that a higher prevalence of HTN, T2DM, OSA, smoking, CHF, and stroke/TIA in NHBs compared to NHWs. Disparities in the rates of AF-related risk factors may account in part for some of the difference observed in population-based studies on the development of AF between NHBs and NHWs. Our data also strengthen findings in the literature suggesting that both H/Ls and NHBs have a higher prevalence of AF-related traditional risk factors, including HTN, T2DM, and smoking compared to NHWs [6], [7].

Previous studies examining cardiovascular risk factors and stroke in H/Ls have demonstrated a strong association of HTN and T2DM with stroke in both sexes, while cigarette smoking is mildly associated with stroke [14]. Our study confirms that HTN and T2DM were strong risk factors contributing to stroke in H/Ls. The prevalence of smoking was significantly higher in H/L men in our study compared to other men, yet it did not significantly increase stroke risk in both sexes.

We also examined the effect of sex on the outcome of stroke. A recent *meta*-analysis revealed that AF is associated with a higher risk stroke and death in women than men [14]. Similarly, our findings showed that females in the total study cohort had lower rates of comorbidities for AF but a higher prevalence of stroke/TIA than men. In terms of sex-specific differences, NHB females with HTN, CAD or COPD had a higher risk of developing stroke, whereas hypertensive H/L females had a marginally higher risk of stroke. H/L females with chronic lung disease, however, had a significantly higher risk of stroke. As for NHW females, smoking increased their risk of stroke. Again, whether these sex-specific differences in AF risk factors may amplify the risk of stroke and worse outcomes of AF requires further investigation.

### 3.7 Limitations

Our study has a number of limitations that should be mentioned. *First,* the patients in this cohort were divided into subgroups based on self-reported ethnicity, which has been shown to be significantly less sensitive in H/Ls as compared to NHWs and NHBs [15]. *Second*, the algorithm used to identify patients with AF is known to be relatively insensitive [16]. It is also important to acknowledge that ICD-9 codes may not accurately identify AF risk factors. *Third*, our study did not adjust for covariates of SES as these have contributed to ethnic disparities in the rates of ischemic stroke among H/Ls as well as potentiate the AF-related risk factors for stroke. *Fourth*, there may be selection bias when relying on EHRs from hospitalized patients with AF. Furthermore, to determine the role of ethnicity in AF etiology would require longitudinal follow-up of an unselected population of subjects. *Fifth*, we were not able to examine differences in treatment modality or quality of care across race-ethnicity; this may have contributed to the increased stroke rates in our minority cohorts. Notably, anticoagulation rates and time in therapeutic range, which have been shown in other studies to differ across race-ethnicity were not assessed [17]. Other treatment modalities have also been shown to differ by race, and were also not captured in this study [7]. *Sixth*, this was a retrospective study with its inherent limitations including failure to estimate the true incidence of AF-related stroke. *Finally,* we did not examine the role of obesity as an AF risk factor in our multi-ethnic cohort. Other AF outcomes such as AF burden, quality of life, and mortality were also not examined.

### 3.8 Conclusions

Although traditional risk factors for the development of AF and its outcomes are well-established in European whites, cohort studies have consistently shown worse AF-related outcomes in ethnic minority groups. We examined the role played by race- and sex-specific risk factors in AF outcomes in NHBs, H/Ls and NHWs and identified differences in risk factors for AF and stroke across race-ethnicity and sex. Our findings are hypothesis generating and should be used to direct future studies.

## Declaration of Competing Interest

The authors declare that they have no known competing financial interests or personal relationships that could have appeared to influence the work reported in this paper.
