# Supplementary material for: Impact of traditional risk factors for the outcomes of atrial fibrillation across race and ethnicity and sex groups
Source: Int J Cardiol Heart Vasc. 2020 May 29;28:100538. doi: 10.1016/j.ijcha.2020.100538 (PMC7262442; doi:10.1016/j.ijcha.2020.100538)
Supplement: Supplementary data 1 [file mmc1.docx]

**Supplementary Table 1. The CHA_2_DS_2_-VASc score**

| **CHADS-VASC scores** | **Non-Hispanic Whites** | **Non-Hispanic Blacks** | **Hispanic/Latinos** |
| --- | --- | --- | --- |
| 0 | 53 (4.91%) | 34 (2.38%) | 12 (3.12%) |
| 1 | 134 (12.42%) | 153 (10.71%) | 29 (7.53%) |
| 2 | 202 (18.72%) | 246 (17.23%) | 49 (12.73%) |
| 3 | 238 (22.06%) | 270 (18.91%) | 83 (21.56%) |
| 4 | 222 (20.57%) | 261 (18.28%) | 75 (19.38%) |
| 5 | 138 (12.79%) | 224 (15.69%) | 68 (17.66%) |
| 6 | 61 (5.65%) | 158 (11.06%) | 40 (10.39%) |
| 7 | 23 (2.13%) | 61 (4.27%) | 22 (5.71%) |
| 8 | 7 (0.65%) | 21 (1.47%) | 7 (1.82%) |
| 9 | 1 (0.09%) | 0 | 0 |
| **Total** | 1079 | 1428 | 385 |

Abbreviations: CHA_2_DS_2_-VASc, congestive heart failure, hypertension, age, diabetes, stroke, vascular, sex.
